# Plasmonic Resonances of Metal Nanoparticles: Atomistic vs. Continuum Approaches

**DOI:** 10.3389/fchem.2020.00340

**Published:** 2020-05-07

**Authors:** Luca Bonatti, Gabriel Gil, Tommaso Giovannini, Stefano Corni, Chiara Cappelli

**Affiliations:** ^1^Scuola Normale Superiore, Piazza dei Cavalieri 7, Pisa, Italy; ^2^Institute of Cybernetics, Mathematics and Physics (ICIMAF), La Habana, Cuba; ^3^Department of Chemical Sciences, University of Padova, Padova, Italy; ^4^Department of Chemistry, Norwegian University of Science and Technology, Trondheim, Norway; ^5^Institute of Nanoscience, National Research Council (CNR), Modena, Italy

**Keywords:** ωFQ, BEM, classical atomistic model, optical spectra, fluctuating charges

## Abstract

The fully atomistic model, ωFQ, based on textbook concepts (Drude theory, electrostatics, quantum tunneling) and recently developed by some of the present authors in *Nanoscale*, **11**, 6004-6015 is applied to the calculation of the optical properties of complex Na, Ag, and Au nanostructures. In ωFQ, each atom of the nanostructures is endowed with an electric charge that can vary according to the external electric field. The electric conductivity between nearest atoms is modeled by adopting the Drude model, which is reformulated in terms of electric charges. Quantum tunneling effects are considered by letting the dielectric response of the system arise from atom-atom conductivity. ωFQ is challenged to reproduce the optical response of metal nanoparticles of different sizes and shapes, and its performance is compared with continuum Boundary Element Method (BEM) calculations.

## 1. Introduction

The study of the plasmonic excitation of metal nanoparticles (NPs) has attracted much interest in the last few decades (Moskovits, 1985; Nie and Emory, 1997; Maier, 2007; Anker et al., 2008; Atwater and Polman, 2010; Santhosh et al., 2016) due to the generation of very strong electric fields in the proximity of their surfaces, which has been exploited in many fields, including for the detection of molecular signals down to the single-molecule limit (Kneipp et al., 1997; Maier et al., 2003; Muehlschlegel et al., 2005; Lim et al., 2010; Giannini et al., 2011; Neuman et al., 2018). Among the plethora of physical features of metal substrates, one of the most relevant is the dependence of their resonance plasmon frequency on the shape, size, and actual material constituting the nanostructure, which permits a fine tuning of the final signal. From a theoretical point of view, the optical properties of nanostructures are generally treated, independently of their size and shape, by resorting to classical approaches (Jin et al., 2001; Hao et al., 2007; Jensen and Jensen, 2008, 2009; Myroshnychenko et al., 2008; Morton and Jensen, 2010, 2011; Pérez-González et al., 2010; Halas et al., 2011; Cirac̀ı et al., 2012; Payton et al., 2012, 2013; Chen et al., 2015; Liu et al., 2017; Mennucci and Corni, 2019), such as the Mie Theory (Mie, 1908), the Discrete Dipole Approximation (DDA) (Draine and Flatau, 1994), and the finite difference time domain methods (FDTD) (Shuford et al., 2006). A viable alternative is to exploit the Boundary Element Method (BEM) (Corni and Tomasi, 2001; de Abajo and Howie, 2002; Hohenester and Trügler, 2012; Hohenester, 2015), in which the NP is treated as a homogeneous, continuum dielectric described by a frequency-dependent permittivity function ε(ω), and the NP surface is modeled as a sharp interface Γ. BEM has been amply applied to reproduce the plasmonic response of NPs of different sizes and shapes, and it has also been extended to take into account quantum tunneling effects, which are relevant for the so-called subnanometer junctions (Esteban et al., 2012).

The wide applicability of the BEM approach is strictly related to its intrinsic low computational cost, which is due to the fact that the NP surface is discretized in terms of point complex charges that interact with the external electric field, giving rise to the resulting polarization. However, when finite size effects, together with edge effects, cannot be neglected, continuum models may fail. In these cases, the atomistic nature of the system needs to be explicitly taken into account. Fully atomistic *ab-initio* approaches, usually based on Density Functional Theory (DFT), address the problem; however, they cannot afford NPs of sizes larger than a few nanometers (hundreds of atoms) due to their high computational cost.

Fully atomistic, yet classical, approaches, able to reproduce both edge and finite size effects and bulk NP properties have been developed so to overcome the limitations of both *ab-initio* and continuum models (Mennucci and Corni, 2019). In particular, classical atomistic modeling of nanoplasmonics has been pioneered by Jensen and coworkers, who developed the Discrete Interaction Model (DIM) (Jensen and Jensen, 2008, 2009; Morton and Jensen, 2010, 2011; Payton et al., 2012, 2013; Chen et al., 2015; Liu et al., 2017). The original version of DIM assigns a frequency-dependent polarizability and a frequency-dependent capacity to each atom, the parameters of which are determined by accurate *ab-initio* calculations (Jensen and Jensen, 2009). However, most DIM applications only exploit the frequency-dependent polarizability (i.e., they neglect capacity terms), thus resulting in an atomistic picture of the Discrete Dipole Approximation (DDA) (Draine and Flatau, 1994; Chen et al., 2015).

An alternative fully atomistic classical model has recently been proposed by some of us (Giovannini et al., 2019e). Such an approach, named ωFQ (frequency-dependent Fluctuating Charges), is based on text-book concepts, i.e., the Drude model for conduction in metals, classical electrostatics, and quantum tunneling (Giovannini et al., 2019e). Each atom of the NP is endowed with an electric charge that is not fixed but can vary as a response to the externally applied oscillating electric field. Thus, ωFQ indeed adopts the atomistic description introduced by Jensen and coworkers, but it sticks to the simplest possible assumptions, e.g., Drude-like conductance even between two (bonded) atoms instead of parameterizing the model based on accurate calculations or empirical inputs (nanoparticle size, neighborhood of the single atom). Notably, ωFQ has been successfully applied to the optical response of subnanometer junctions, where quantum tunneling plays a crucial role. In this work, we apply the model to reproduce the optical properties of single NPs with different shapes and sizes.

The manuscript is organized as follows. In the next section, the main physical features of both ωFQ and BEM are briefly recalled and compared. Then, the computational methodology is presented, and the numerical results are discussed. In particular, ωFQ is tested against the reproduction of the absorption cross-sections of differently shaped Na, Ag, and Au NPs, and its performance is compared with BEM results so as to highlight the similarities and differences of the two models. A section focusing on conclusions and future perspectives for the approach ends the presentation.

## 2. Materials and Methods

### 2.1. Theoretical Models

In this section, the main features of the atomistic ωFQ and continuum BEM approaches are briefly recalled. In particular, the working equations of the two models are presented, and the conceptual differences between the two are discussed in detail.

#### 2.1.1. ωFQ

ωFQ is an atomistic approach aimed at describing the optical properties of a metal NP in the quasi-static limit. ωFQ has its theoretical foundation on the Fluctuating Charges (FQ) force field, which, combined with a QM description of a molecular solute, is usually exploited in the modeling of the spectroscopic properties of solvation phenomena (Cappelli, 2016; Giovannini et al., 2018, 2019b). In the FQ force field, each classical atom is described in terms of a charge, which value can vary as a response of the external sources. Similarly to FQ, in ωFQ, each NP atom is modeled as a charge that varies as a response to an external oscillating electric field [**E**(ω)]. Remarkably, from the mathematical point of view, ωFQ charges are complex because the metal response includes a dissipative part. In particular, their imaginary part is directly related to the absorption cross-section (vide infra).

The equation of motion of ωFQs is written in terms of the Drude model of conductance (Bade, 1957), adequately reformulated in case of charges (Giovannini et al., 2019e):

(1) $$\begin{array}{l} \frac{dq_{i}}{dt}=2n_{0}\underset{j}{\sum }A_{ij}<\text{p}>\cdot {\hat{l}}_{ji} \end{array}$$

where *A*_*ij*_ is the effective area dividing atom *i* by atom *j* and *A*_*ij*_ is a model parameter optimized to reproduce reference *ab-initio* data. *n*_0_ is the atomic electron density, < **p** > is the momentum of an electron averaged over all trajectories connecting *i* and *j*, and ${\hat{l}}_{ji}=-{\hat{l}}_{ij}$ is the unit vector of the line connecting *j* to *i*. By replacing **p** with its expression in terms of the external electric field **E**(ω) within the Drude model (Giovannini et al., 2019e), Equation (1) can be rewritten in the frequency domain as:

(2) $$\begin{array}{l} -i\omega q_{i}=2n_{0}\underset{j}{\sum }A_{ij}\frac{<\text{E}\left(\omega \right)>\cdot {\hat{l}}_{ji}}{1/\tau -i\omega } \end{array}$$

where τ is a friction-like constant due to scattering events. By then assuming $<\text{E}\left(\omega \right)>\cdot {\hat{l}}_{ji}\approx \left(\mu_{j}^{el}-\mu_{i}^{el}\right)/l_{ij}$, where $\mu_{i}^{el}$ is the electrochemical potential of atom *i* and *l*_*ij*_ the distance between atoms *i* and *j*, Equation (2) becomes:

(3) $$\begin{array}{l} -i\omega q_{i}=\frac{2n_{0}}{1/\tau -i\omega }\underset{j}{\sum }\frac{A_{ij}}{l_{ij}}\left(\mu_{j}^{el}-\mu_{i}^{el}\right)\\ \;=\underset{j}{\sum }\left[\frac{2\sigma_{0}/\tau }{1/\tau -i\omega }\frac{A_{ij}}{l_{ij}}\right]\left(\mu_{j}^{el}-\mu_{i}^{el}\right)\\ \;=\underset{j}{\sum }K_{ij}^{\text{dru}}\left(\mu_{j}^{el}-\mu_{i}^{el}\right) \end{array}$$

where *n*_0_ = σ_0_/τ, with σ_0_ being the static conductance of the considered metals. In Equation (3), a matrix named *K*^dru^ with elements $K_{ij}^{\text{dru}}=\frac{2n_{0}}{1/\tau -i\omega }\frac{A_{ij}}{l_{ij}}$ has been introduced.

Equation (3) describes the electron transfer between all atoms constituting the metal NP. However, in order to make the model physically consistent, i.e., to avoid electron transfer between atoms that are too far apart, in ωFQ, the pairs of atoms in Equation (3) are limited to nearest neighbors only. In order to impose such a limitation, a Fermi-like *f*(*l*_*ij*_) damping function is introduced to weight the Drude conductive mechanism:

(4) $$\begin{array}{l} -i\omega q_{i}=\underset{j}{\sum }\left(1-f\left(l_{ij}\right)\right)\cdot K_{ij}^{\text{dru}}\left(\mu_{j}^{el}-\mu_{i}^{el}\right)\\ \;=\underset{j}{\sum }K_{ij}^{\text{tot}}\left(\mu_{j}^{el}-\mu_{i}^{el}\right) \end{array}$$

where:

(5) $$\begin{array}{l} f\left(l_{ij}\right)=\frac{1}{1+\exp\left[-d\left(\frac{l_{ij}}{s\cdot l_{ij}^{0}}-1\right)\right]} \end{array}$$

In Equation (5), $l_{ij}^{0}$ is the equilibrium distance between two nearest neighbors, whereas *d* and *s* are parameters determining the position of the inflection point and the steepness of the curve. It is worth noticing that due to the exponential decay of the Fermi-like function in Equation (5) (Esteban et al., 2012; Giovannini et al., 2019e), ωFQ is also capable of treating the quantum tunneling that governs the electron transfer between the atoms in the NP (Giovannini et al., 2019e).

Finally, Equation (4) can be rewritten by defining the electrochemical potential μ in terms of the external potential *V*^ext^:

(6) $$\begin{array}{l} \;\underset{j}{\sum }\left(-\underset{k}{\sum }K_{ik}^{\text{tot}}D_{ij}+\underset{k}{\sum }K_{ik}^{\text{tot}}D_{kj}+i\omega \delta_{ij}\right)q_{j}\\ =\underset{j}{\sum }\left(V_{i}^{\text{ext}}-V_{j}^{\text{ext}}\right)K_{ij}^{\text{tot}} \end{array}$$

where *D*_*ij*_ is the electrostatic coupling kernel written in terms of Gaussian charges (Mayer, 2007; Giovannini et al., 2019c,e) and δ_*ij*_ is the Kronecker delta. Equation (6) finally gives the complex ωFQs charges from which the complex polarizability $\bar{\alpha }$ and thus the absorption cross-section σ^abs^ can be recovered:

(7) $$\begin{array}{l} \bar{\alpha }{\left(\omega \right)}_{kl}=\frac{\partial {\bar{\mu }}_{k}\left(\omega \right)}{\partial E_{l}\left(\omega \right)}=\underset{i}{\sum }q_{i}\left(\omega \right)\cdot \frac{k_{i}}{E_{l}\left(\omega \right)}\\ \;\Rightarrow \;\sigma^{\text{abs}}=\frac{4\pi }{3c}\omega \text{ tr}\left(\text{Im}\left(\bar{\alpha }\left(\omega \right)\right)\right) \end{array}$$

where μ is the complex dipole moment, *i* runs over NP atoms, *k* represents the *x, y, z* positions of the *i*-th atom, and *l* runs over the *x*,*y*,*z* directions of the external electric field. *c* is the speed of light, and $\text{Im}\left(\bar{\alpha }\right)$ is the imaginary part of the complex polarizability $\bar{\alpha }$.

ωFQ has recently been developed and applied to describe the optical response of sodium (Na) nanoparticles (Giovannini et al., 2019e). In fact, due to the specificity of the model, it describes the conductive electrons only by neglecting any possible contribution arising from *d*-electrons. Nevertheless, the model can be used in its present form to model the optical properties of any metal at frequencies that are far from interband transitions, i.e., those dominated by *d*-electrons.

#### 2.1.2. Continuum Dielectric Model and Boundary Element Method

The Boundary Element Method (BEM) is a classical electrodynamics approach in which the NP is treated as a homogenous, continuum dielectric described by a frequency-dependent permittivity function ε(ω), and the NP surface is modeled as a sharp interface Γ (Fuchs, 1975; Corni and Tomasi, 2001; de Abajo and Howie, 2002; Vukovic et al., 2008; Hohenester and Trügler, 2012; Angioni et al., 2013; Mennucci and Corni, 2019). In this work, BEM quasi-static formulation, valid whenever the size of the NP is much smaller than the wavelength of the incident light, is exploited.

Under the action of an external oscillating electric field **E**(ω), a surface charge density, which mimics the polarization, arises on the nanostructure. From the computational point of view, this electrodynamical phenomenon is solved by discretizing the NP surface by an ensemble of tesserae derived from the surface discretization. As a consequence, the surface charge density is modeled in terms of a set of point charges **q**, which are called Apparent Surface Charge (ASCs) (Corni and Tomasi, 2001; de Abajo and Howie, 2002; Hohenester and Trügler, 2012). The BEM equation for solving the ASCs per tessera reads:

(8) $$\begin{array}{l} \text{q}\left(\omega \right)=-{\left[2\pi \left(\frac{\varepsilon \left(\omega \right)+1}{\varepsilon \left(\omega \right)-1}\right){\text{A}}^{-1}+{\text{D}}^{†}\right]}^{-1}{\text{E}}_{n}\left(\omega \right) \end{array}$$

where **E**_*n*_(ω) is the normal component of the applied electric field calculated at each charge position, whereas **A** is a diagonal matrix containing the tessera areas. **D** is a matrix that is defined in terms of the tesserae positions **s**_*i*_ and the outgoing normal unit vector per tessera **n**_*i*_ as:

(9a) $$\begin{array}{l} D_{ij}=\frac{\left({\text{s}}_{i}-{\text{s}}_{j}\right)\cdot {\text{n}}_{j}}{|{\text{s}}_{i}-{\text{s}}_{j}{|}^{3}}\text{ for }i\neq j\;, \end{array}$$

(9b) $$\begin{array}{l} D_{ii}=2\pi -\underset{j\neq i}{\sum }D_{ij} \end{array}$$

BEM Equation (8) depends on the NP geometry (**D** matrix) and material (ε(ω)). It is worth noticing that Equation (8) formulates the optical response of NPs in vacuo. However, the generalization for any surrounding dielectric medium is straightforward and can be achieved by replacing Λ = (ε(ω) + 1)/(ε(ω) − 1) by Λ = (ε(ω) + ε_*ext*_(ω))/(ε(ω) − ε_*ext*_(ω)) in Equation (8), where ε_*ext*_(ω) is the dielectric function of the external medium (Corni et al., 2001; Corni and Tomasi, 2002). Similarly to ωFQ, ACSs are complex quantities because dielectric functions are complex themselves.

Finally, the absorption cross-section σ^abs^ is recovered from the definition of the complex polarizability ${\bar{\alpha }}_{ij}$, which in turn is defined in terms of the complex dipole moment $\bar{\mu }$:

(10) $$\begin{array}{l} \bar{\mu }\left(\omega \right)=\overset{T}{\underset{k=1}{\sum }}q_{k}\left(\omega \right){\text{s}}_{k}\;\Rightarrow \;{\bar{\alpha }}_{ij}\left(\omega \right)=\frac{\mu_{i}\left(\omega \right)}{E_{j}\left(\omega \right)}\\ \;\Rightarrow \;\sigma_{\text{abs}}=\frac{4\pi }{3c}\omega \;\text{tr}\left(\text{Im}\left(\bar{\alpha }\left(\omega \right)\right)\right) \end{array}$$

where *k* runs over the number of tesserae, whereas *i* and *j* indicate Cartesian components.

### 2.2. Computational Details

**Figure 1:**
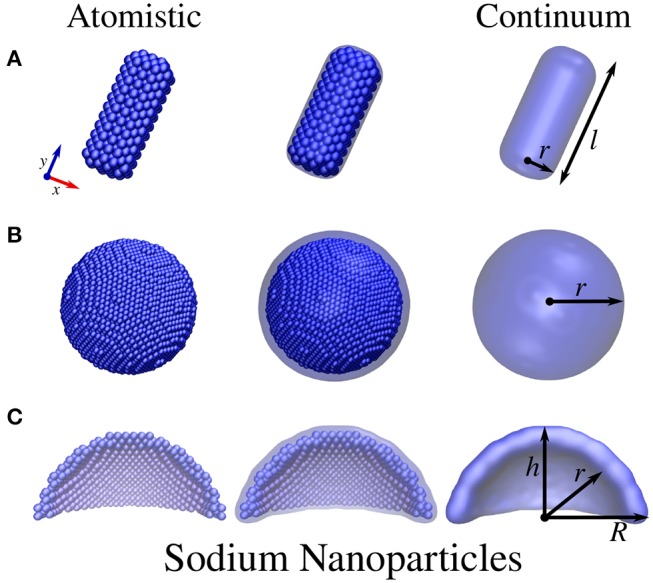
Atomistic (left) and continuum (right) sodium NP structures. **(A)** Cylindrical nanorod with length *l* and radius *r*. **(B)** Spherical NP with radius *r*. **(C)** Spherical nanodome with internal radius *r*, external radius *R*, and dome height *h*. The dome has been cut in half so as to best represent the structure.

**Figure 2:**
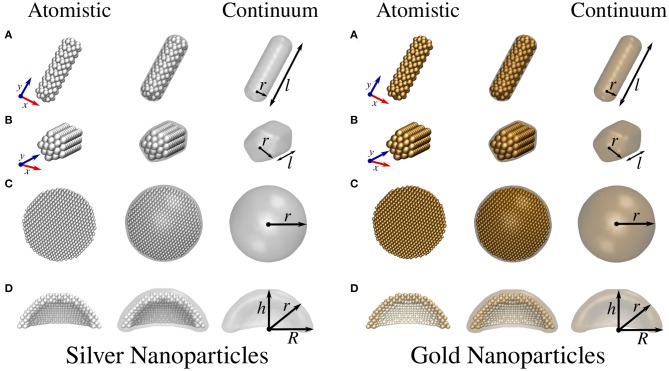
Atomistic (left) and continuum (right) silver and gold NP structures. **(A)** Cylindrical nanorod with length *l* and radius *r*. **(B)** Pentagonal nanorod with length *l* and radius *r*. **(C)** Spherical NP with radius *r*. **(D)** Spherical nanodome with internal radius *r*, external radius *R*, and dome height *h*. The dome has been cut in half so as to best represent the structure.

Atomistic ωFQ and continuum BEM approaches were challenged to reproduce the optical response of Na, Ag, and Au nanoparticles of different shapes, varying from cylindrical and pentagonal nanorods and spherical nanodomes (see Figures 1, 2 for NP structures).

The geometries of atomistic Na nanorods (see Figure 1A) were constructed by imposing a cylindrical section (with radius *r* and length *l*) and Na lattice constant equal to 4.23 Å (Haynes, 2014) in a Body-Centered Cubic (BCC) packing. The geometries of atomistic Ag and Au nanorods (see Figures 2A,B) were instead created by imposing both a cylindrical and a pentagonal section (with radius *r* and length *l*) and Ag/Au lattice constant equal to 4.08 Å (Haynes, 2014) in a Face-Centered Cubic (FCC) packing. The geometries of Na, Ag, and Au spherical nanodomes (see Figures 1C, 2D) were constructed by removing a semi-sphere of radius *r* from a concentric semi-sphere of radius *R* > *r* (see Figures 1B, 2C), retaining the same packing and distances mentioned above in the case of nanorods. Different heights of the obtained dome were considered by retaining all atoms that are placed at a certain distance *h* from the top of the dome.

All atomistic structures exploited for the following ωFQ calculations were obtained by using the Atomic Simulation Environment (ASE) Python module v. 3.17 (Larsen et al., 2017). The numbers of atoms in Na, Ag, and Au structures are reported in Tables S1–S8 (Supplementary Material).

The continuum structures for the same geometries described above were constructed by using the MNPBEM Matlab toolbox (v. 17) (Hohenester and Trügler, 2012). In particular, cylindrical nanorods are constructed out of a cylinder with two hemispheres covering the flat circular faces at its extremes. Analogously, pentagonal nanorods are build up from an extruded pentagon and two pentagonal hemispheres covering the flat pentagonal faces at the extremes. The pentagonal hemispheres are obtained by considering hemispheres discretized with five meridians, so as to generate an object with a pentagonal cross-section normal to the long axis of the nanorod. The pentagonal nanorods were smoothened out at the edges. The full tessellation of such nanorods resulted in 798 and 2212 tesserae, respectively. Spherical shells are built from two concentric spheres (see Figures 1B,C, 2C,D), the inner of which is void. In the case of spherical nanodome portions, the full tessellation contains 600 tesserae.

ωFQ cross-sections were calculated on the obtained atomistic structures by using a stand-alone Fortran 95 package that is under development by some of the present authors. Equation (6) is solved for a set of frequencies given as input. All computed spectra reported in the manuscript were obtained by explicitly solving linear response equations for steps of 0.01 eV. For all studied Na and Ag nanosystems, the ωFQ parameters defined in Equations 3–4 were taken from Giovannini et al. (2019e). The parameters for Au nanostructures are instead reported here for the first time and were recovered from literature data where available: τ = 3.2·10^−14^ s (Palik, 1997), $\sigma_{0}=2.4\cdot 10^{7}$ S/m (Haynes, 2014), *A*_*ij*_ = 3.38 Å^2^, $l_{ij}^{0}=2.88$ Å (Haynes, 2014), *d* = 12.00, and *s* = 1.10 (Giovannini et al., 2019e). ωFQ Na, Ag, and Au parameters are given in Tables S9–S11 (Supplementary Material).

BEM cross-sections were computed by using the MNPBEM Matlab toolbox (v. 17) (Hohenester and Trügler, 2012). Similarly to ωFQ simulations, Equation (8) was solved for steps of 0.01 eV. The dielectric functions defined in Equation (8) were recovered from experimental data (Rakić et al., 1998 for Ag and Au and Althoff and Hertz, 1967; Smith, 1969 for Na).

Both ωFQ and MNPBEM simulations were limited to the quasi-electrostatic (non-retarded) approximation because the size of the largest studied nanoparticle (nanorods with *r* = 50 Å and *l* = 150 Å) is much smaller than the computed absorption wavelength.

## 3. Results and Discussion

In this section, ωFQ and BEM absorption cross-sections (σ^abs^) of sodium, silver, and gold NPs of different shapes (see Figures 1, 2) are presented and compared so as to highlight the differences arising by exploiting atomistic/continuum approaches. The presentation of the computed results is divided into three sections, the first presenting a comparison between both ωFQ and BEM and *ab-initio* σ^abs^ for silver/gold pentagonal nanorods. The second and third sections are instead focused on Na NPs and Ag and Au NPs, respectively. This way of presenting the results is justified by the following: (i) the comparison with *ab-initio* results permits a quantitative analysis of the performance of the ωFQ and BEM approaches; (ii) Ag and Au are characterized by FCC packing, whereas Na has BCC packing, which makes the number of atoms of the structures rapidly incomparable; (iii) the Ag and Au structures are identical because they are characterized by the same lattice constant; (iv) both Ag and Au present inter-band transitions, whereas Na has a simpler dielectric response (interband transitions are, however, present above 2 eV).

### 3.1. ωFQ and BEM Benchmarking

**Figure 3:**
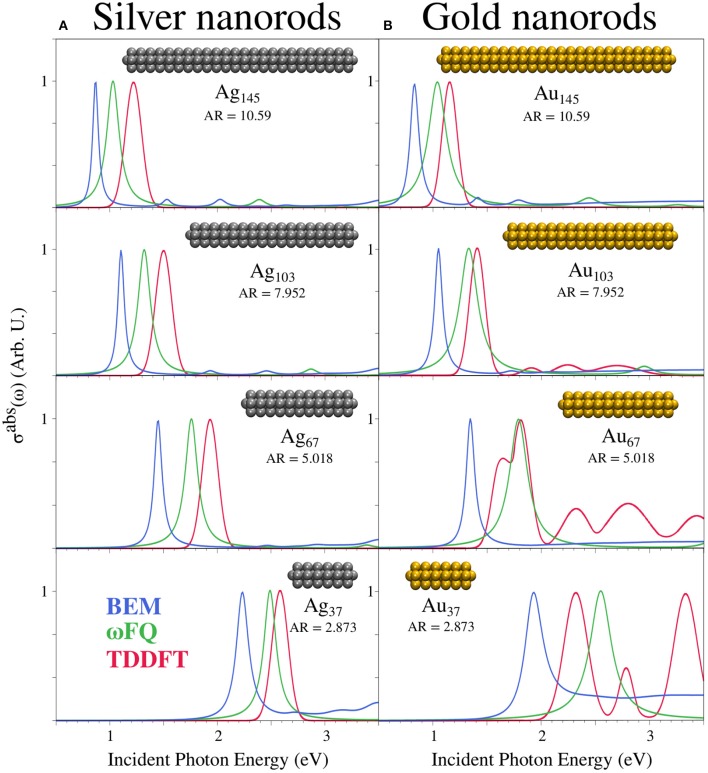
BEM(blue), ωFQ (green), and TD-DFT (red) absorption cross-sections σ^abs^ of silver **(A)** and gold **(B)** pentagonal nanorods with different numbers of atoms (see labels). TD-DFT data are reproduced from Sinha-Roy et al. (2017). The aspect ratio (AR) of the selected nanorods is also given.

In Figure 3, ωFQ and BEM σ^abs^ of silver/gold pentagonal nanorods (see Figure 2B) constituted by a different number of atoms (see labels) and r=2.8 Å are reported together with reference TD-DFT results reproduced from Sinha-Roy et al. (2017). As can be seen, all ωFQ and BEM results are characterized by an intense peak that redshifts as the length of the nanorod increases. Such a trend is perfectly in agreement with the reference data. In addition, the ωFQ Plasmon Resonance Frequencies (PRFs) are in better agreement with the corresponding TD-DFT values than are those of BEM, independently of the length of the nanorod. Such findings suggest that for these structures, which are characterized by atomically defined edges, ωFQ is more reliable than BEM. We also remark that the well-known explanation of the blueshift of small nanoparticles of noble metal was devised for spherical metal nanoparticles (Liebsch, 1993) whose resonance frequency is near to the interband absorption edge. There, the *d*− electron screening is enhanced by the pre-resonance condition. The nanorods considered here have their resonance far from that region, and the shifts are dominated by atomistic effects that are grasped well by ωFQ.

### 3.2. Sodium Nanoparticles

**Figure 4:**
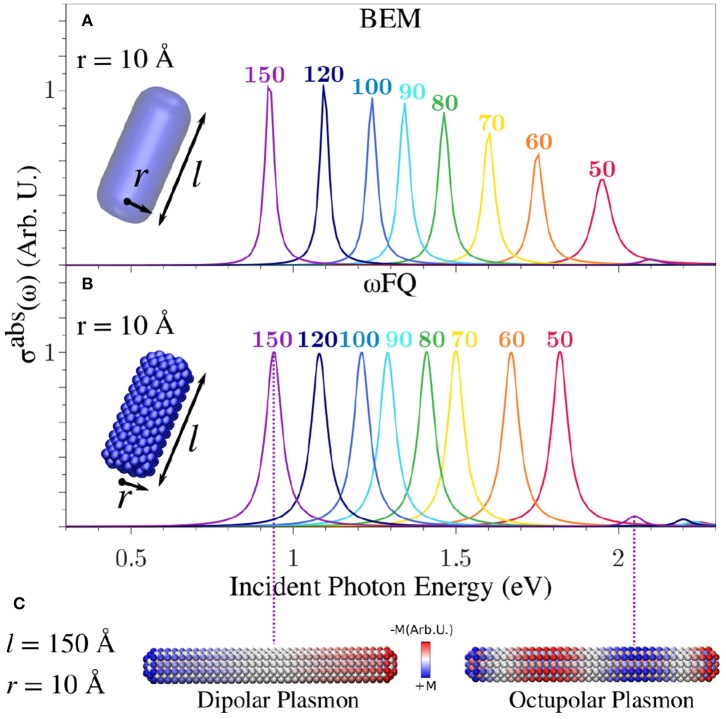
Calculated BEM **(A)** and ωFQ **(B)** absorption cross-sections σ^abs^ of a sodium nanorod with *r* = 10 Å as a functions of *l* (see labels). **(C)** ωFQ imaginary charges for a Na nanorod with *r* = 10 Å and *l* = 150 Å calculated at the PRFs of the two peaks highlighted in **(B)**. All σ^abs^ are normalized with respect to the volume of the considered structure.

Figure 4 presents BEM (Figure 4A) and ωFQ (Figure 4B) σ^abs^ of Na cylindrical nanorods with radius *r* = 10 Å as a function of the length *l*. BEM and ωFQ intensities were normalized with respect to nanorod volumes and NP numbers of atoms, respectively. The calculated absorption cross-sections are characterized by intense and sharp peaks, independently of the nanorod length and the exploited model (BEM or ωFQ). The nature of the plasmon associated to each peak is highlighted by plotting ωFQ imaginary charges calculated at the PRF for a sample structure with *l* = 150 Å (see Figure 4C). It is clear that the main band is related to a Boundary Dipolar Plasmon (BDP), which, remarkably, is in line with previous studies (Rossi et al., 2015; Sinha-Roy et al., 2017; Giovannini et al., 2019e).

Both BEM and ωFQ PRF redshift by increasing the length of the metal nanorod, in agreement with classical electrodynamics. By deepening in the comparison between the BEM and ωFQ results, we notice that, despite the trends being almost identically reproduced by both models, some deviations appear in the cases of the shortest nanorods (see Figure 4). This is probably due to the fact that finite-size effects cannot be neglected by decreasing the length of the nanorod. In addition, the PRF for such small structures shifts above 2 eV, thus indicating that interband transitions may play a relevant role. Also, it is worth noticing that ωFQ intensities remain constant in all spectra, whereas BEM intensities decrease as the length of the nanorod decreases, with a simultaneous increase in the band broadening (Kreibig and Fragstein, 1969), which is not reported by ωFQ. In order to better understand the reasons for these discrepancies, the areas of all peaks have been computed and normalized with respect to the area of the peak of the longest nanorod (l = 150 Å, see Figure S1). We notice that all of the ωFQ peak areas almost perfectly match the BEM data, thus showing that the physics of the system is similarly described by the two approaches. It is worth remarking that an increase in the band broadening as the length of the nanorod decreases has been reported previously in the literature (Kreibig and Fragstein, 1969). Such an effect is correctly modeled by BEM, due to the fact that it exploits the experimental permittivity function. These features are not reproduced by ωFQ; however, several *ad-hoc* techniques, which have been developed in the past (Kreibig and Fragstein, 1969; Liebsch, 1993; Molina et al., 2002; Gao et al., 2011) to solve such issues, could in principle be coupled with our approach.

The general agreement between ωFQ and BEM in the cases of the largest structures is also confirmed by the presence of a second peak in the high-frequency region (see e.g., *l* = 150 Å at ~2.1 eV in Figure 4). The nature of such a band, which is very low in intensity, was investigated by plotting ωFQ imaginary charges similarly to the most intense band. From inspection of Figure 4C, the peak can be easily related to a boundary octupolar plasmon, confirming what has been reported in the literature (Rossi et al., 2015; Giovannini et al., 2019e). We remark that some mixing with the dipolar plasmon is needed to see σ^abs^≠ 0.

**Figure 5:**
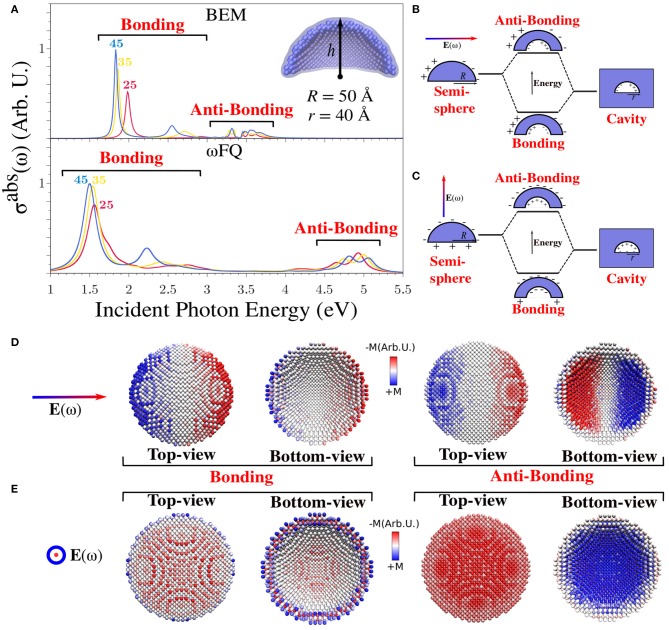
**(A)** BEM (top) and ωFQ (bottom) isotropic absorption cross-sections σ^abs^ of different sodium spherical nanodomes with *r* = 40 Å and *R* = 50 Å as a function of the height (*h*) of the dome (25 < *h* < 45 Å). **(B,C)** Graphical depiction of the model exploited to explain the plasmonic character of the absorption peaks with co-axial and orthogonal polarizations. **(D,E)** ωFQ imaginary charges for a Na nanodome with *r* = 40 Å, *R* = 50 Å, and *h* = 45 Å calculated with co-axial and orthogonal polarizations at the PRFs of the two peaks (bonding and anti-bonding) highlighted in **(A)**. Top and bottom views of the dome are depicted. All σ^abs^ are normalized with respect to the volume of the considered structure.

To further investigate the performance of ωFQ and BEM, we selected a challenging system, i.e., a portion of a spherical Na (see Figure 1C), which has not been widely investigated before from both the theoretical and experimental points of view (Ye et al., 2009; Raja et al., 2016). In Figure 5A, the absorption cross-section of such structure as a function of the height *h* of the dome is reported. Notice that the internal *r* and external *R* radii are kept fixed to 40 and 50 Å, respectively. Three main features, common to both ωFQ and BEM absorption spectra, can be highlighted.

- All spectra are characterized by an intense and sharp peak at about 1.5 eV (ωFQ) and 1.8 eV (BEM) that redshifts by increasing the height of the dome (i.e., by moving from *h*=25 to *h*=45 Å). Such a trend is in agreement with what has been shown above in the case of nanorods (see Figure 4). In fact, increasing the height of the dome results in an increase in the number of atoms, which, as in the previous case, results in a redshift of the absorption spectrum. Differently from nanorods, BEM and ωFQ PRFs differ by almost 0.3 eV; this is probably due to a different description of the nanodome edges.
- All spectra present a second low-in-intensity peak in the region between 2 and 3 eV. Both the intensities and PRFs of such bands show the same trends already commented on for the first intense peak.
- Finally, two additional bands arise for all structures in the region between 3 and 4 eV (BEM) and 4-5 eV (ωFQ). It is worth noticing that for such a composite band, the trend reproduced by ωFQ PRFs is opposite with respect to the low-in-energy peaks, i.e., PRFs blueshift as the structures are enlarged. BEM PRFs are instead almost constant with an increase in the system's size.

In order to physically investigate these findings, imaginary ωFQ charges were depicted and rationalized in terms of the plasmon hybridization approach (Prodan et al., 2003a,b; Radloff and Halas, 2004; Wang et al., 2006a,b, 2007; Bardhan et al., 2009; Park and Nordlander, 2009). In particular, spherical nanodome plasmonic response can be represented by analyzing a simple model in which such a geometry is seen as resulting from the difference between a section of spherical NP and a section of a spherical cavity in an infinite Na structure (see Figure 5B). The direct consequence of such a model is that the plasmons arising in the nanodome can be viewed as the linear combination of the plasmons arising in the two pristine structures. In fact, the antisymmetric and symmetric combinations give rise to two plasmonic modes, which are named bonding and anti-bonding, respectively (see Figures 5B,C). Notice also that the studied nanodomes have cylindrical symmetry. Therefore, an electric field parallel or orthogonal to the symmetry axis activates three different plasmonic excitations. Two of them will be degenerate, i.e., where the external field is orthogonal to the symmetry axis. The two alternatives are graphically depicted in Figures 5B,C.

The main features of Na nanodome spectra can be rationalized in terms of the above simple model. In fact, the two excitations at lower energy can be related to bonding modes, as is confirmed by the graphical representation of the imaginary ωFQ charges calculated at PRFs (see Figures 5D,E, in which the two possible orientations of the electric field are considered). It is worth noticing that the two bonding modes are qualitatively different; in the case where the electric field is orthogonal to the symmetry axis (see Figure 5D), a BDP appears both in the external and in the internal surface of the shell, whereas in the other case (see Figure 5E), the basis of the shell is the only portion that is positively charged, and the external surface is globally negatively charged.

The two peaks at high energies (see Figure 5A) can instead be associated to the anti-bonding modes, which are placed at different PRFs depending on the direction of the external electric field. This is the reason why each computed spectrum is characterized by the presence of two peaks. Similarly to bonding modes, also in this case, we characterized the nature of the plasmonic modes by plotting the imaginary ωFQ charges calculated at PRFs (see Figures 5D,E). If the electric field is orthogonal to the symmetry axis, the anti-bonding mode is characterized by an opposite BDP on the internal and the external surfaces, exactly as predicted by the simple model in Figure 5B. In the orthogonal field polarization, such a mode is instead characterized by the internal surface negatively charged and the external positively charged, and the basis of the shell is almost zero charged. Again, all the present features can entirely be explained by the suggested model.

To conclude the discussion on Na nanodomes, it is worth remarking that Figure 5A reports isotropic cross-sections. Therefore, the bonding and antibonding modes result in a pair of peaks because co-axial and orthogonal polarizations are not degenerate. This can be further appreciated by looking at Figure S2 in Supplementary Material.

### 3.3. Silver and Gold Nanoparticles

**Figure 6:**
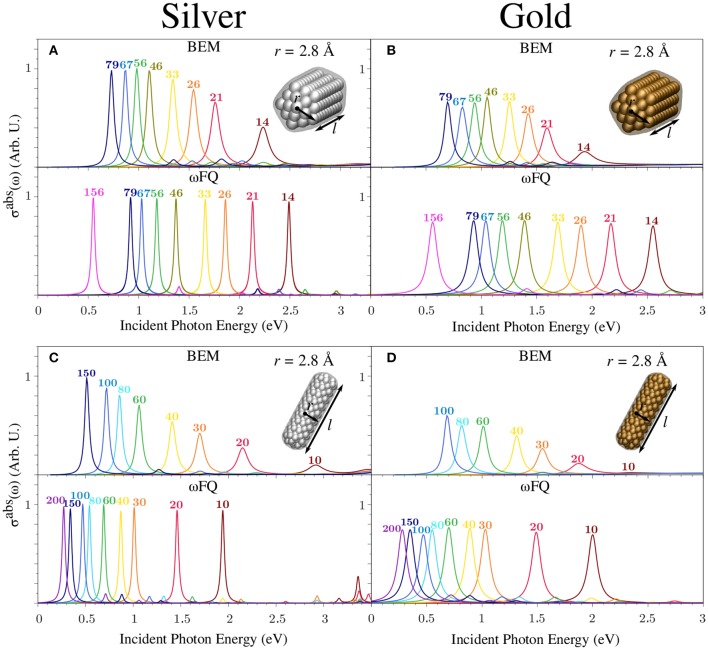
BEM (top) and ωFQ (bottom) absorption cross-sections σ^abs^ of pentagonal **(A,B)** and cylindrical **(C,D)** silver (left) and gold (right) nanorods with *r* = 2.8 Å as a function of *l* (see labels). All σ^abs^ are normalized with respect to the volume of the considered structure.

Figure 6 presents BEM (top) and ωFQ (bottom) σ^abs^ of silver (left) and gold (right) pentagonal (Figures 4A,B) and cylindrical (Figures 4C,D) nanorods with radius *r* = 2.8 Å as a function of the length *l* (see Figures 2A,B for their structures). Similarly to the case of Na NPs, BEM and ωFQ intensities were normalized with respect to nanorod volumes and the number of NP atoms, respectively.

The ωFQ-calculated absorption cross-section of Ag nanorods (Figure 6, bottom, left) in both the considered geometries (i.e., pentagonal and circular section) is characterized by a sharp and intense peak independently of the length of the nanorod. Such a peak is related to BDP as in the case of Na nanorods (see Figure 4). A similar outcome is shown by Au NPs (Figure 6, bottom, right) in both geometrical arrangements, but in this case, the absorption band is much broader and lower in intensity. This is in agreement with previous work (Sinha-Roy et al., 2017) and is an expected consequence of the choice of ωFQ parameters, in particular τ, which in Au NPs is almost three times lower than in Ag NPs (see Equation 3 and Tables S9–S11). In both Ag and Au NPs, ωFQ computed PRFs redshift as the length of the nanorod is increased, again in agreement with previous work (Sinha-Roy et al., 2017) (see also Figure 3). We remark that Ag and Au ωFQ PRFs are in much better agreement with the corresponding *ab-initio* data reported by Sinha-Roy et al. (2017) (see Figure 3), thus confirming the validity of the proposed parametrization and the potentialities of ωFQ to describe the plasmonic character of noble metals, at least far from the inter-band region (Giovannini et al., 2019e). In addition, as already noticed in the case of Na nanorods (see Figure 4), each ωFQ spectrum is characterized by a second low-in-intensity peak at higher energies with respect to the most intense one, which can once again be related to high-order plasmons (octupolar, Rossi et al., 2015; Giovannini et al., 2019e).

Similarly to ωFQ, BEM Ag σ^abs^ (Figure 6, top, left) is characterized by a single absorption band whose maximum redshifts, shrinks, and increases in intensity as the nanorod is elongated. Also, BEM spectra show an additional peak at higher energies, which is again related to octupolar plasmons. The same trend is also reported in the case of Au nanorods (Figure 6, top, right), which also exhibits a general decrease in the intensity that is in agreement with the ωFQ results. Deepening the comparison between ωFQ and BEM, the agreement between the two approaches increases as larger structures are considered. Such an agreement is not only due to the reproduction of PRFs but also to a general qualitative reproduction of the entire spectrum. Remarkably, such a finding might be justified by considering that small structures (*l*≲26 Å) are probably characterized by non-negligible edge effects, which cannot be modeled by continuum approaches such as BEM. In addition, we remark that the Drude function that is adopted in ωFQ (see Equation 3) does not consider possible interband transitions. Therefore, the observed discrepancies may also be connected to the differences between the experimental permittivity function ε(ω) (exploited in BEM) and the description given by the Drude model. We also notice that in the case of pentagonal nanorods, for both Ag and Au, BEM PRFs are redshifted with respect to the corresponding ωFQ values, whereas the opposite occurs in the case of cylindrical geometries. For pentagonal nanorods, this is in agreement with what has already been discussed in section 3.1. The reported discrepancies between ωFQ and BEM can be ascribed to the different geometrical arrangements of the nanorods. In fact, in the case that the geometry of the NP is characterized by the presence of edges (for BEM) or isolated atomic chains (for ωFQ), a redshift is expected. This is confirmed by comparing the BEM results for pentagonal (edges) and cylindrical (no edges) nanorods (Figures 6A,C top, respectively) and the ωFQ PRFs for pentagonal (no presence of atomic chains) and cylindrical (presence of atomic chains) nanorods (Figures 6A,C bottom, respectively). Therefore, the aforementioned differences between BEM and ωFQ are basically due to the fact that the BEM structures in Figures 6A,B are characterized by the presence of edges, whereas ωFQ structures do not have atomic chains. The opposite is seen in Figures 6C,D. Furthermore, we remark that the atomistic structures exploited in ωFQ are unambiguously determined by the lattice constant of the studied material. In BEM, in contrast, different approximations need to be done to represent a given atomistic structure (smooth/sharp edges, capping, etc.), and this constitutes a limitation of the BEM approach itself.

**Figure 7:**
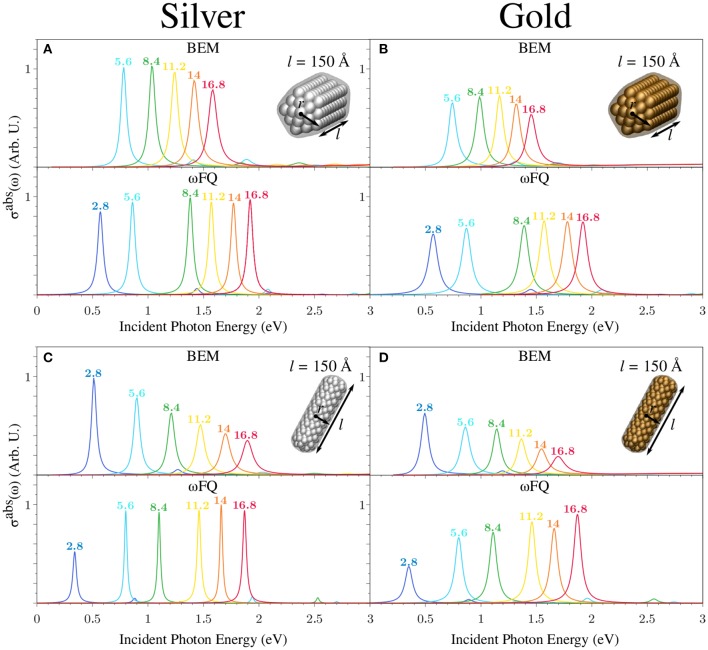
BEM (top) and ωFQ (bottom) absorption cross-sections σ^abs^ of pentagonal **(A,B)** and cylindrical **(C,D)** silver (left) and gold (right) nanorods with *l* = 150 Å as a function of *r* (see labels). All σ^abs^ are normalized with respect to the volume of the considered structure.

Let us now focus on calculated ωFQ (bottom) and BEM (top) σ^abs^ of a set of Ag and Au nanorods with fixed length *l* (150 Å) as a function of the radius *r* in both pentagonal and cylindrical shapes (see Figure 7). The comments presented above for Figure 6 still hold. In fact, all ωFQ Ag absorption spectra are characterized by an intense and sharp peak followed by another low-in-intensity peak at higher energies. The former is once again associated to a BDP, whereas the latter has an octupolar character. The most appreciable differences between Ag and Au spectra are associated with the smaller intensities and the larger broadening in the case of Au, as already commented on in the previous case. The same considerations applied to ωFQ results can be extended to BEM. It is worth noticing that, differently to the previous case (see Figure 6), the two approaches are nicely in agreement in reproducing PRFs of both Ag and Au cylindrical nanorods. Such an outcome can be explained by considering that the structures reported in Figure 7 are much larger than those studied in Figure 6; thus, edge effects are expected to play a minor role. This, however, does not occur in the case of pentagonal nanorods, which show almost the same trend as that already discussed above for smaller nanorods. Such an outcome can be justified by the fact that BEM structures are affected by edge effects. In fact, BEM PRFs are redshifted with respect to BEM cylindrical nanorods (see Figures 7A–C, top). ωFQ PRFs for cylindrical/pentagonal nanorods are instead almost identical for the largest nanorods (*r* ≥ 11.2 Å), thus confirming that edge effects become more and more negligible as the radius of the nanorod increases.

**Figure 8:**
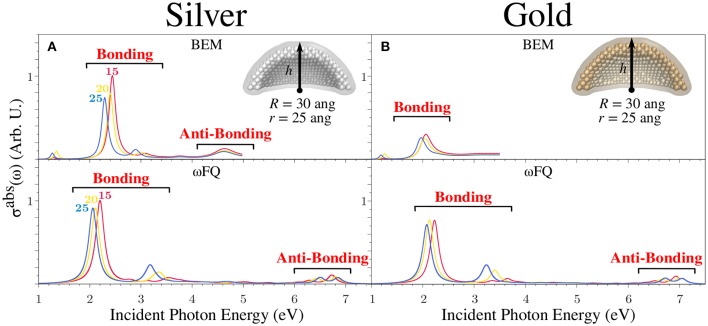
BEM (top) and ωFQ (bottom) isotropic absorption cross-sections σ^abs^ of silver **(A)** and gold **(B)** spherical nanodomes with *r* = 25 Å and *R* = 30 Å as a function of *h* (15 < *h* < 25 Å). All σ^abs^ are normalized with respect to the volume of the considered structure.

To conclude the discussion on Ag and Au NPs, we studied the absorption cross-section for a spherical nanodome with *r* = 25 Å and *R* = 30 Å as a function of the height of the dome, similarly to what we also reported in case of Na NPs. The results are graphically depicted in Figures 8A,B for Ag and Au structures, respectively. The outcomes already discussed in the case of Na nanodomes are, for the most part, confirmed. In fact, for both metals, σ^abs^ is characterized by an intense pair of peaks between 2 and 3 eV, of which the PRF redshifts as the NP size increases. Again, such bands are related to bonding plasmonic modes showing charge distributions almost identical to those depicted in Figures 5D,E (see also Figure S2). Notice that also in this case, the two polarizations (i.e., co-axial and orthogonal) give rise to two non-degenerate absorption peaks due to the symmetry breaking that has been introduced by cutting the pristine sphere in half (see also the Computational Details Section). In addition, ωFQ predicts a pair of two high-energy peaks, which can instead be related to antibonding plasmonic modes. Notice that the latter bands undergo a blueshift as the NP number of atoms increases, thus resulting in an opposite behavior with respect to that underlined in the case of the bonding modes. Finally, it is worth noticing that the discussed antibonding excitations are not fully reported for BEM. This is due to the fact that the experimental permittivity function (ε(ω)) adopted to model the plasmonic response of the two metals (see Equation 8) is not defined in the considered energy range.

## 4. Conclusions

In this work, the potentialities of the atomistic ωFQ approach to describe the optical properties of sodium, silver, and gold NPs characterized by different geometrical arrangements have been investigated. The results have been compared with BEM, and theoretical analogies and differences between ωFQ and BEM have been discussed and analyzed in terms of the physical quantities underlying both approaches. The main difference between ωFQ and BEM lies in the fact that in the former, the atomistic nature of the NP is retained, whereas in the latter, the NP is modeled through its surface only. Despite such differences, both models describe the polarization in terms of complex charges, which are placed on each NP atom in the case of ωFQ, whereas, in BEM, they are located at points defined through the discretization procedure of the NP surface.

ωFQ and BEM have been applied to Na, Ag, and Au cylindrical and pentagonal (in the case of Ag and Au NPs) nanorods and spherical nanodomes. The results obtained with the two methods are nicely in agreement for Na nanorods, whereas some discrepancies are present for Na spherical nanodomes. For the latter, to explain the different plasmonic modes that arise in the absorption spectrum, the plasmon hybridization model has been applied (Bardhan et al., 2009; Park and Nordlander, 2009). In particular, both ωFQ and BEM predict bonding and anti-bonding plasmonic modes, which have been highlighted by plotting imaginary charges arising on the NP surfaces. Remarkably, the breaking of the spherical symmetry in the studied structures gives rise to two different non-degenerate plasmonic modes depending on the polarization of the incident electric field, i.e., co-axial or perpendicular to the symmetry axis. It is also worth noticing that some discrepancies are present in ωFQ and BEM PRFs of the different plasmonic modes, which may be related to edge effects and to interband transitions [which, in the case of Na, occur above 2 eV (Smith, 1969)].

Similar results have been obtained for Ag and Au structures. In the case of cylindrical and pentagonal nanorods, the discrepancies between ωFQ and BEM PRFs tend to disappear with an increase in the size of the studied structures. The results obtained with the two methods are similar in the case of metal nanodomes, for which bonding and anti-bonding plasmonic modes are correctly described by ωFQ. Antibonding excitations have not been studied by BEM, because the experimental permittivity function (ε(ω)) of the two metals is not available in the considered energy range. Although this drawback can be easily overcome by modeling the permittivity function with a Drude model, the quality of BEM results would inevitably lose accuracy due to the inaccurate description of interband transitions.

To conclude, our results show that the novel ωFQ atomistic approach can effectively describe the optical properties of metal NPs far from the energy range of interband transitions. This limitation can, however, be overcome by introducing in the ωFQ approach the physical features that are needed to correctly account for *d*-electrons in noble metals, e.g., by including an additional term in the response equations expressed in terms of an atomic dipole, similarly to what has recently been done by some of us in a different context (Giovannini et al., 2019a,c,d). Such an extension is currently under development and will be a topic of future communications.

## Data Availability Statement

The datasets generated for this study are available on request to the corresponding author.

## Author Contributions

LB ran the ωFQ calculations. GG ran the BEM calculations. TG wrote the stand-alone Fortran95 code for ωFQ calculations. TG, LB, and GG analyzed data and wrote the manuscript. TG, SC, and CC discussed and supervised the whole project. All authors revised and checked the draft.

## Conflict of Interest

The authors declare that the research was conducted in the absence of any commercial or financial relationships that could be construed as a potential conflict of interest.
