# Supplementary material for: Plasmonic Resonances of Metal Nanoparticles: Atomistic vs. Continuum Approaches
Source: Front Chem. 2020 May 7;8:340. doi: 10.3389/fchem.2020.00340 (PMC7221199; doi:10.3389/fchem.2020.00340)
Supplement: Supplementary file 1 [file Data_Sheet_1.PDF]

Supplementary Material

1 SUPPLEMENTARY DATA

2 NANOPARTICLES' GEOMETRIES

Element	NP geometry	l [Å]	r [Å]	Atoms
Na	cylindrical rod	50	10	428
Na	cylindrical rod	60	10	515
Na	cylindrical rod	70	10	613
Na	cylindrical rod	80	10	687
Na	cylindrical rod	90	10	786
Na	cylindrical rod	100	10	872
Na	cylindrical rod	120	10	1045
Na	cylindrical rod	150	10	1292

Table S1. Geometrical parameters and number of atoms for sodium cylindrical nanorods (see Fig. 1A). l is the length of the rod whereas r is its radius.

Element	NP geometry	R [Å]	r [Å]	h [Å]	Atoms
Na	spherical shell	50	40	25	1542
Na	spherical shell	50	40	35	2158
Na	spherical shell	50	40	45	2942

Table S2. Geometrical parameters and number of atoms for sodium spherical shells (see Fig. 1C). R and r are the external and internal radii, respectively, whereas h is the height of the dome.

Element	NP geometry	l [Å]	r [Å]	Atoms
Ag	cylindrical rod	16	2.8	10
Ag	cylindrical rod	26	2.8	20
Ag	cylindrical rod	44	2.8	30
Ag	cylindrical rod	55	2.8	40
Ag	cylindrical rod	66	2.8	50
Ag	cylindrical rod	76	2.8	60
Ag	cylindrical rod	94	2.8	70
Ag	cylindrical rod	104	2.8	80
Ag	cylindrical rod	126	2.8	100
Ag	cylindrical rod	186	2.8	150
Ag	cylindrical rod	246	2.8	200
Ag	cylindrical rod	150	5.6	820
Ag	cylindrical rod	150	8.4	1952
Ag	cylindrical rod	150	11.2	3910
Ag	cylindrical rod	150	14.0	6008
Ag	cylindrical rod	150	16.8	8974

Table S3. Geometrical parameters and number of atoms for silver cylindrical nanorods (see Fig. 2A, left). l is the length of the rod whereas r is the radius.

Element	NP geometry	l [Å]	r [Å]	Atoms
Ag	pentagonal rod	14	2.8	37
Ag	pentagonal rod	21	2.8	49
Ag	pentagonal rod	26	2.8	61
Ag	pentagonal rod	33	2.8	73
Ag	pentagonal rod	46	2.8	97
Ag	pentagonal rod	53	2.8	109
Ag	pentagonal rod	56	2.8	121
Ag	pentagonal rod	63	2.8	133
Ag	pentagonal rod	67	2.8	145
Ag	pentagonal rod	73	2.8	157
Ag	pentagonal rod	79	2.8	169
Ag	pentagonal rod	150	2.8	313
Ag	pentagonal rod	156	2.8	325
Ag	pentagonal rod	150	5.6	855
Ag	pentagonal rod	150	8.4	2655
Ag	pentagonal rod	150	11.2	4057
Ag	pentagonal rod	150	14.0	5587
Ag	pentagonal rod	150	16.8	7619

Table S4. Geometrical parameters and number of atoms for silver pentagonal nanorods (see Fig. 2B, left). l is the length of the rod whereas r is the radius.

Element	NP geometry	R [Å]	r [Å]	h [Å]	Atoms
Ag	spherical shell	30	20	15	617
Ag	spherical shell	30	20	20	917
Ag	spherical shell	30	20	25	1137

Table S5. Geometrical parameters and number of atoms for silver spherical shells (see Fig. 2D, left). R and r are the external and internal radii, respectively, whereas h is the height of the dome.

Element	NP geometry	l [Å]	r [Å]	Atoms
Au	cylindrical rod	16	2.8	10
Au	cylindrical rod	26	2.8	20
Au	cylindrical rod	44	2.8	30
Au	cylindrical rod	55	2.8	40
Au	cylindrical rod	66	2.8	50
Au	cylindrical rod	76	2.8	60
Au	cylindrical rod	94	2.8	70
Au	cylindrical rod	104	2.8	80
Au	cylindrical rod	126	2.8	100
Au	cylindrical rod	186	2.8	150
Au	cylindrical rod	246	2.8	200
Au	cylindrical rod	150	5.6	820
Au	cylindrical rod	150	8.4	1952
Au	cylindrical rod	150	11.2	3910
Au	cylindrical rod	150	14.0	6008
Au	cylindrical rod	150	16.8	8974

Table S6. Geometrical parameters and number of atoms for gold cylindrical nanorods (see Fig. 2A, right). l is the length of the rod whereas r is the radius.

Element	NP geometry	l [Å]	r [Å]	Atoms
Au	pentagonal rod	14	2.8	37
Au	pentagonal rod	21	2.8	49
Au	pentagonal rod	26	2.8	61
Au	pentagonal rod	33	2.8	73
Au	pentagonal rod	46	2.8	97
Au	pentagonal rod	53	2.8	109
Au	pentagonal rod	56	2.8	121
Au	pentagonal rod	63	2.8	133
Au	pentagonal rod	67	2.8	145
Au	pentagonal rod	73	2.8	157
Au	pentagonal rod	79	2.8	169
Au	pentagonal rod	150	2.8	313
Au	pentagonal rod	156	2.8	325
Au	pentagonal rod	150	5.6	855
Au	pentagonal rod	150	8.4	2655
Au	pentagonal rod	150	11.2	4057
Au	pentagonal rod	150	14.0	5587
Au	pentagonal rod	150	16.8	7619

Table S7. Geometrical parameters and number of atoms for gold pentagonal nanorods (see Fig. 2B, right). l is the length of the rod whereas r is the radius.

Element	NP geometry	R [Å]	r [Å]	h [Å]	Atoms
Au	spherical shell	30	20	15	617
Au	spherical shell	30	20	20	917
Au	spherical shell	30	20	25	1137

Table S8. Geometrical parameters and number of atoms for gold spherical shells (see Fig. 2D, right). textitR and r are the external and internal radii, respectively, whereas h is the height of the dome.

3 NA, AG AND AU ωFQ PARAMETERS

Parameter	Eq. (main text)	Value [a.u.]	Ref
η	3	0.292	(Giovannini et al., 2019)
τ	3	1323	(Palik, 1998)
σ_0	3	5.21	(Gall, 2016)
A_{ij}	3	12.08	(Giovannini et al., 2019)
d	5	100.0	(Giovannini et al., 2019)
s	5	1.2	(Giovannini et al., 2019)
l^0_{ij}	5	6.92	(Haynes, 2014)

Table S9. ωFQ parameters for Na NPs used in this paper. All data are given in atomic units.

Parameter	Eq. (main text)	Value [a.u.]	Ref
η	3	0.379	(Giovannini et al., 2019)
τ	3	1200	(Palik, 1998)
σ_0	3	13.7	(Haynes, 2014)
A_{ij}	3	12.079	(Giovannini et al., 2019)
d	5	100.0	(Giovannini et al., 2019)
s	5	0.92	(Giovannini et al., 2019)
l^0_{ij}	5	5.44	(Sinha-Roy et al., 2017)

Table S10. ωFQ parameters for Ag NPs used in this paper. All data are given in atomic units.

Parameter	Eq. (main text)	Value [a.u.]	Ref
η	3	0.417624	t.w.
τ	3	890.22	(Gall, 2016)
σ_0	3	9.83	(Gall, 2016)
A_{ij}	3	15.9576	t.w.
d	5	100.0	t.w.
s	5	0.92	t.w.
l^0_{ij}	5	5.44	(Sinha-Roy et al., 2017)

Table S11. ωFQ parameters for Au NPs used in this paper (t.w.: this work). All data are given in atomic units.

4 PEAK AREAS OF FIG. 3 OF THE MAIN TEXT

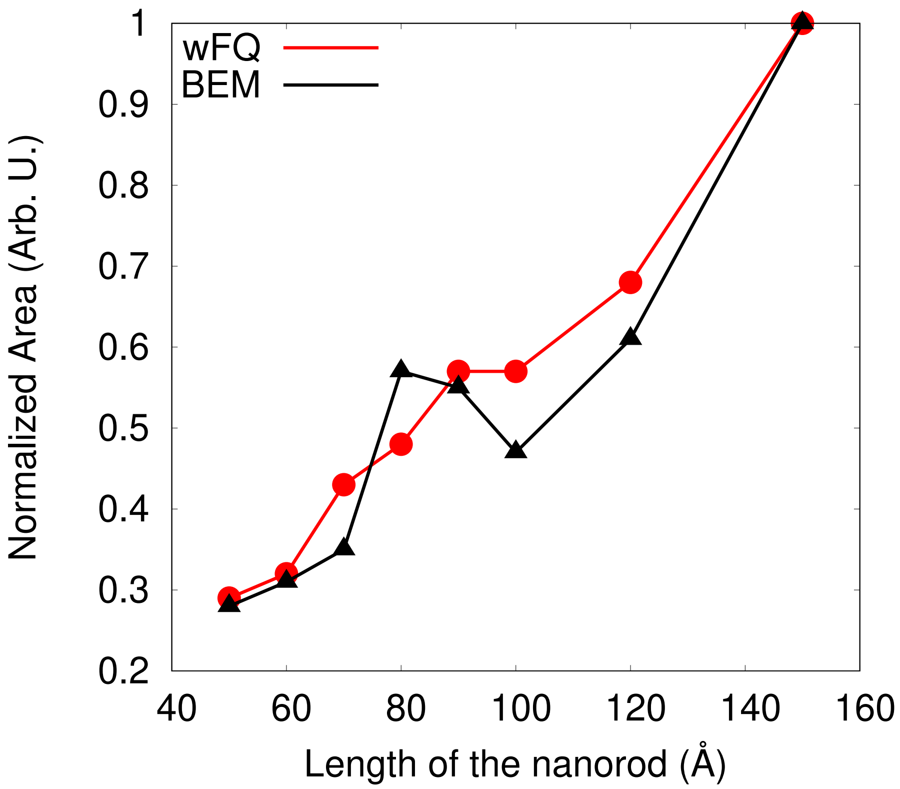

Figure S1. Calculated ωFQ and BEM peak areas of Na nanorods reported in Fig. 3 of the main text. All areas are normalized with respect to the longest nanorod (150 Å).

5 ωFQ ABSORPTION CROSS SECTION OF NA NANOSHELLS

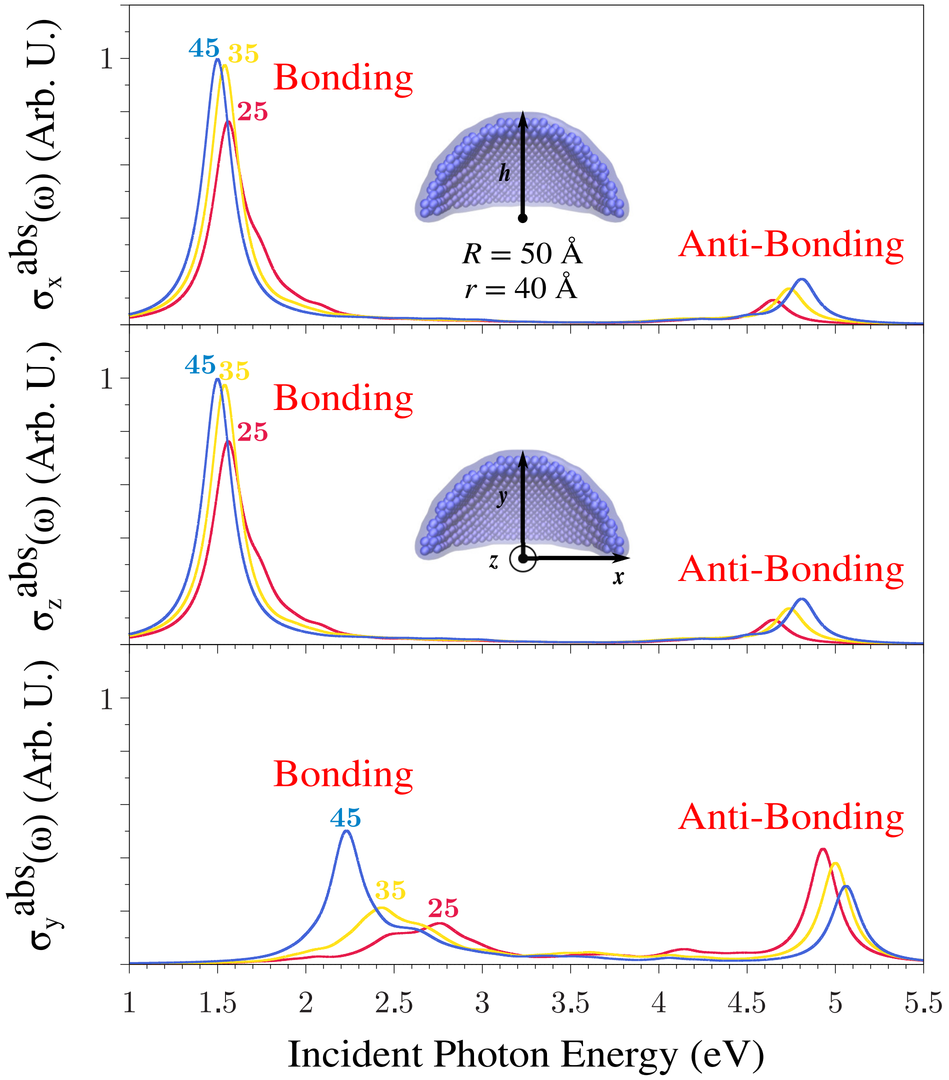

Figure S2. Calculated ωFQ absorption cross section σ_{abs} of Na spherical nanoshells (r = 40 Å and R = 50 Å) as a function of the height (h) of the dome ($25 < h < 45$ Å). The three different external polarization are plotted: x (top), z (middle), y (bottom).

REFERENCES

Gall, D. (2016). Electron mean free path in elemental metals. *Journal of Applied Physics* 119, 085101

Giovannini, T., Rosa, M., Corni, S., and Cappelli, C. (2019). A classical picture of subnanometer junctions: an atomistic drude approach to nanoplasmonics. *Nanoscale* 11, 6004–6015

Haynes, W. M. (2014). *CRC handbook of chemistry and physics* (CRC press)

Palik, E. D. (1998). *Handbook of optical constants of solids*, vol. 3 (Academic press)

Sinha-Roy, R., García-González, P., Weissker, H.-C., Rabilloud, F., and Fernandez-Dominguez, A. I. (2017). Classical and ab initio plasmonics meet at sub-nanometric noble metal rods. *ACS photonics* 4, 1484–1493
